# Side Effects and Interactions of the Xanthine Oxidase Inhibitor Febuxostat

**DOI:** 10.3390/ph11020051

**Published:** 2018-05-25

**Authors:** Andreas Jordan, Ursula Gresser

**Affiliations:** Internal Medicine, Medical Faculty, Ludwig Maximilians University of Munich, 80539 Munich, Germany; jordan.andreas@gmx.de

**Keywords:** febuxostat, side effects, interactions

## Abstract

The paper addresses the safety of febuxostat and summarizes reports on side effects and interactions of febuxostat published by the cut-off date (last day of literature search) of 20 March 2018. Publications on side effects and the interactions of febuxostat were considered. Information concerning the occurrence of side effects and interactions in association with the treatment with febuxostat was collected and summarized in the review. The incidence of severe side effects was much less frequent than mild side effects (1.2–3.8% to 20.1–38.7%). The rate and range of febuxostat side effects are low at doses of up to 120 mg and only increase with a daily dose of over 120 mg. The publications reveal no age-dependent increase in side effects for febuxostat. In patients with impaired renal function, no increase in adverse events is described with a dose of up to 120 mg of febuxostat per day. Patients with impaired liver function had no elevated risk for severe side effects. A known allopurinol intolerance increases the risk of skin reactions during treatment with febuxostat by a factor of 3.6. No correlation between treatment with febuxostat and agranulocytosis has been confirmed. Possible interactions with very few medications (principally azathioprine) are known for febuxostat. Febuxostat is well tolerated and a modern and safe alternative to allopurinol therapy.

## 1. Introduction

**Figure 1:**
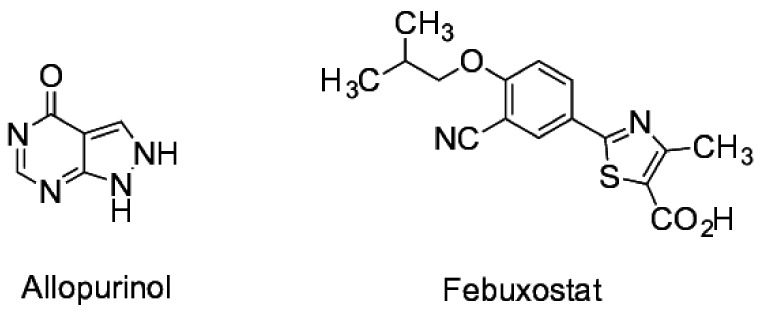
Chemical structures of allopurinol and febuxostat [3].

Allopurinol (market launch in Germany 1964, [1]) and febuxostat (market launch in Germany 2010, [2]) are two inhibitors of the xanthine oxidase. Febuxostat is, other than allopurinol, a non-purine xanthine oxidase inhibitor (see Figure 1) [3].

Spiekermann showed, that the xanthine oxidase is also located in the vessel wall [4]. There is evidence for a connection between the activity of xanthine oxidase and vasodilation as well as endothelial function [5]. The free oxygen radicals formed during xanthine oxidase play an important pathophysiological role in this context [6]. It was demonstrated that—in contrast to allopurinol—febuxostat has a positive impact on stress parameters and vascular elasticity [7].

The present review addresses the following questions:

1. What is known from scientific publications with regard to side effects and interactions during treatment with the xanthine oxidase inhibitor febuxostat?
2. Febuxostat’s safety profile in comparison and contrast to allopurinol.

## 2. Results

### 2.1. Findings in Original Works and Secondary Analyses

Table A1 and Table A2 present the original works and secondary analyses with the respective events that occurred.

The results of the evaluation of the stated publications are summarized below by content. The administered daily febuxostat dose in the reviewed papers was between 10 mg and 240 mg.

In the original papers and secondary analyses, at least one side effect (without severe side effects) occurred in 20.1% and 39.7% of the patients and at least one severe side effect in 1.2% and 3.8%, respectively. The five most frequently reported side effects were musculoskeletal symptoms (7.7% of the patients who received febuxostat); upper respiratory tract symptoms (5.4%); changes in liver function values (4.7%); diarrhea (3.6%); headache (2.8%). Of the patients in the original papers and secondary analyses, 2.9% and 1.0%, respectively, dropped out of the study prematurely due to side effects. The most frequent side effect reported in the original papers that resulted in a patient dropping out of the study prematurely was an increase in liver values (20.9%) [8,9,10,11,12]. Other reasons were diarrhea (12.0%), rash (1.7%), as well as cardiac symptoms (1.7%). The latter were three patients with unspecified cardiovascular symptoms [9], one patient with precordial pain [13], one patient with palpitations and chest pain [14], as well as one patient with acute heart failure [15]. In the CARES-study from White et al., the incidence of major cardiovascular events was similar in both groups [16]. “Sudden cardiac death was […] occuring in […] 2.7% [of the patients] in the febuxostat group and […] 1.8% [of the patients] in the allopurinol group” [16]. The risk of cardiovascular death was higher under febuxostat therapy compared to allopurinol in patients with gout [16]. The overall mortality in both groups was similar [16].

#### 2.1.1. Duration of therapy and dosage

**Figure 2:**
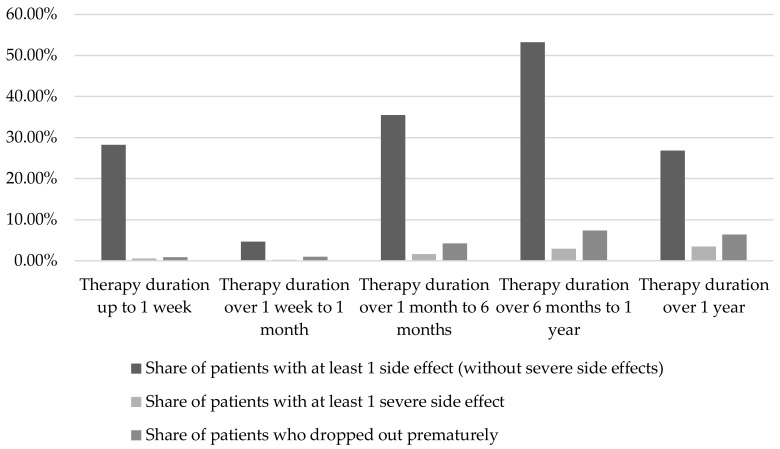
Occurrence of adverse events in dependence on therapy duration (n (therapy duration up to one week) = 332; n (therapy duration over one week to one month) = 6525; n (therapy duration over one month to six months) = 3170; n (therapy duration over six months to one year) = 1297; n (therapy duration over one year) = 917; per therapy period differentiation between patients with at least one side effect (column 1), patients with at least one severe side effect (column 2), patients who dropped out of the study prematurely (column 3)) [2,8,9,10,11,12,13,14,15,17,18,19,20,21,22,23,24,25,26,27,28,29,30,31,32,33,34,35,36,37,38,39,40,41,42,43,44,45,46,47,48,49,50,51,52].

An increase in the occurrence of events with the increasing duration of the therapy was shown for severe side effects (see Figure 2). The share of patients affected increased from 0.6% (up to one week) to 3.5% (more than one year). The share of patients with at least one side effect (without severe side effects) was in a range between 4.7% (duration of therapy over one week to one month) and 53.3% (duration of therapy over six months to one year) during the therapy periods considered. The share of these patients in the periods considered were the lowest for a duration of therapy over one week to one month (4.7%) and over one year (26.9%). The share of patients who dropped out of the study prematurely due to side effects increased from 0.9% (duration of therapy up to 1 week) to 6.4% (duration of therapy of more than one year).

**Figure 3:**
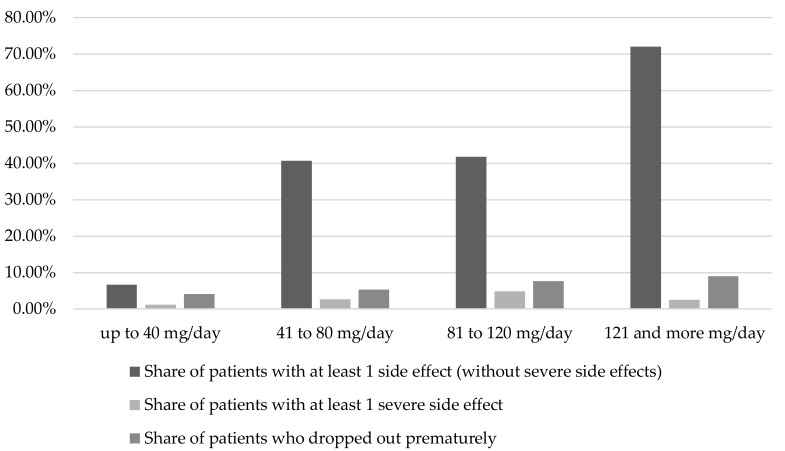
Occurrence of adverse events in dependence on febuxostat dosage (n (up to 40 mg/day) = 1803; n (41 to 80 mg/day) = 2361; n (81 to 120 mg/day) = 673; n (121 and more mg/day) = 200; for each dose group differentiation between: patients with at least one side effect (column 1), patients with at least one severe side effect (column 2), patients who dropped out prematurely (column 3); daily dose used in mg/day for each dose group: up to 40 mg/day: 10, 20, 30, 40; 41 to 80 mg/day: 50, 60, 70, 80; 81 to 120 mg/day: 90, 120; 121 mg/day and more: 160, 180, 240) [8,9,11,12,13,15,17,18,19,20,21,22,23,24,25,26,27,29,31,33,34,35,36,38,41,42,45,48,49,50,51,52,53,54].

As the dose increased, the share of patients with at least one side effect (without severe side effects) was 72% at a daily dose of febuxostat of more than 120 mg (see Figure 3). At lower doses, this share was a maximum of 41.8%. In the dose group of 81 to 120 mg/day compared with the dose group of 51 to 80 mg/day, an increase in the share of patients with at least one severe side effect due to febuxostat from 2.6% to 4.8% was observed. The share of patients who dropped out of the study prematurely increased to a maximum of 9% in the highest dose group (121 and more mg/day).

A quantification of the interaction between the duration of therapy and the dose was not possible based on the information provided in the original studies.

#### 2.1.2. Renal Function

**Figure 4:**
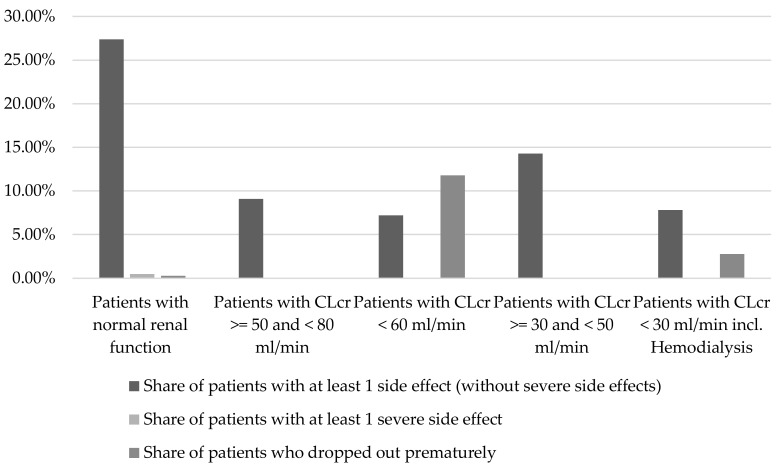
Occurrence of adverse events depending on renal function (n (patients with normal renal function) = 1812; n (patients with CLcr >= 50 and <80 mL/min) = 11; n (patients with CLcr <60 mL/min) = 610; n (patients with CLcr >=30 and <50 mL/min) = 35; n (patients with CLcr <30 mL/min incl. hemodialysis) = 399; per patient group (sorted by renal function, left starting with the best) differentiation between patients with at least one side effect (column 1), patients with at least one severe side effect (column 2), patients who dropped out prematurely (column 3)) [12,13,14,17,18,19,20,22,24,25,26,29,31,34,35,37,38,41,44,45,46,48,49,50,51,52,55].

The share of patients with impaired renal function, who experienced at least one side effect (without severe side effects) was a maximum of 14.3% and thus lower than for patients with normal renal function (27.4%) (see Figure 4). The maximum febuxostat dose for patients with impairment in renal function was at 80 mg/day, lower than for patients with normal renal function (maximum 240 mg/day).

#### 2.1.3. Liver Function

The metabolisation of febuxostat in the body largely takes place in the liver [50]. In the study by Khosravan et al., the subjects were allocated into three groups based on the individual hepatic function as determined by Child-Pugh [50]. In patients with moderately impaired liver function, the share of patients with at least one side effect (without severe side effects) was 75% and therefore three times as high as in the patient group with normal liver function (25%) [50] (see Table 1). The daily dose of febuxostat in all groups was 80 mg [50]. The most frequently reported side effects (without severe side effects) were headache, abdominal pain, and diarrhea, as well as a change in the frequency of micturition [50]. It was not necessary to adjust the febuxostat dose in any of the cases [50]. No severe side effects occurred in any of the groups [50].

#### 2.1.4. Diabetes mellitus type 2

**Figure 5:**
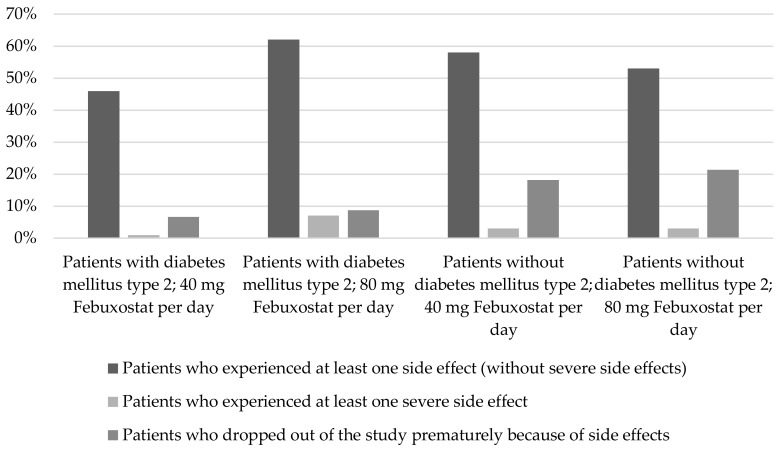
Occurrence of adverse events in patients with and without diabetes mellitus type 2 (n (Study population) = 1462; classification of patients by patients with and without diabetes mellitus type 2 as well as respective febuxostat dose (40 or 80 mg/day); in each case, illustration of patients who experienced at least one side effect (column 1), patients who experienced at least one severe side effect (column 2), patients who dropped out of the study prematurely (column 3)) [56].

The occurrence of adverse events during treatment with febuxostat in patients with and without diabetes mellitus type 2 is comparable based on the publications reviewed (see Figure 5) [39,56].

In Ito et al. [39], at least one side effect (without severe side effects) occurred among 13.5% of the diabetes patients treated with febuxostat. Among patients without diabetes mellitus type 2 the share was 20% [39].

**Figure 6:**
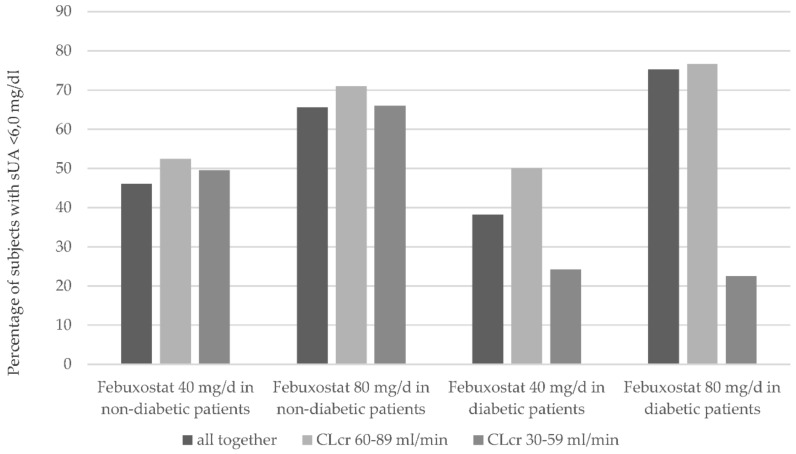
Comparing diabetic and non-diabetic patients and the urate lowering efficiency of febuxostat (patients with achievement of serum urate level < 6.0 mg/dL in percent) at a daily dose of febuxostat of 40 mg/day respectively 80 mg/day with regard to the renal function level (sUA: serum urate level) [56].

Becker et al. [56] showed that the urate lowering efficiency of febuxostat (reducing the serum urate level below 6 mg/dL) is dose dependent in both patients with and without renal insufficiency. Febuxostat at a daily dose of 80 mg was more efficient than 40 mg at any level of renal insufficiency (see Figure 6). This holds true for both diabetic and non-diabetic patients [56].

#### 2.1.5. Age Dependency

Studies indicated an increase in adverse events in patients over 65 years of age [21,57]. The share of patients who experienced at least one side effect (without severe side effects) and one severe side effect was twice as high in the group of patients over the age of 65 (58.3% and 6.3%) than in the group of patients under 65 years (29.2% and 2.5%) [57]. The number of comorbidities in the study population of the patients under 65 years was in part only half as high as in the group of patients over 65 [57]. In the younger group every patient took an average of 3.8 medications versus 7.1 medications in the older patient group [57].

#### 2.1.6. Pre-existing Allopurinol Intolerance

Based on the reviewed publications, the risk of a skin reaction (exanthem, pruritus) is higher by a factor of 3.6 under therapy with febuxostat in patients with a pre-existing allopurinol intolerance [28].

#### 2.1.7. Combination of Febuxostat with NSAIDs

Non-steroidal anti-inflammatory drugs (NSAIDs) are frequently used to treat acute attacks of gout. The incidence of side effects was investigated during simultaneous therapy with febuxostat together with Indometacin or Naproxen (see Table 2) [52].

Based on the publications reviewed regarding the side effect profile, Indometacin and Naproxen can be prescribed parallel to a therapy with febuxostat to the extent that there is no contraindication for NSAID therapy in a given case [52].

#### 2.1.8. Combination of Febuxostat with Hydrochlorothiazide

In the therapy of febuxostat together with hydrochlorothiazide, 43% of the patients developed at least one side effect [12]. This share was 41% when the only treatment given was febuxostat [12]. Severe side effects did not occur [12]. The combined therapy of febuxostat and hydrochlorothiazide was well tolerated [12]. No dose adjustment is required for the simultaneous administration of febuxostat and hydrochlorothiazide [12].

#### 2.1.9. Combination of Febuxostat with Uricosuric Drugs

Lesinurad (approved in Europe) and Arhalofenat (not yet approved in Europe) are drugs that increase the excretion of uric acid in the urine and are used to treat hyperuricemia and gout [58,59]. In 21 patients treated with febuxostat (40 or 80 mg/day) together with Lesinurad (400 to 600 mg/day), a total of 27 side effects and no severe side effects occurred [58]. During the combined use of febuxostat (40 and 80 mg/day) and Arhalofenat (600 or 80 mg/day) in 32 patients, 23 patients (72%) experienced at least one side effect and no severe side effects [59].

#### 2.1.10. Combination of Febuxostat with Azathioprine

Two cases of a pancytopenia or eosinophilia have been published to date in response to a possible interaction between febuxostat and azathioprine [55,60]. Both authors consider an interaction of these two medications as the probable source of the symptoms [55,60]. Patients experienced, among other adverse reactions, nausea and vomiting, watery diarrhea with weight loss, as well as pancytopenia [55]; other manifestations included fever and a case of eosinophilia [60].

#### 2.1.11. Combination of Febuxostat with Theophylline

The coadministration of febuxostat together with theophylline does not affect the pharmacokinetics of theophylline [24]. Theophylline was well tolerated when administered together with febuxostat (80 mg/day) without dose adjustment of febuxostat [24].

### 2.2. Findings of the Case Reports

Research revealed 14 published case reports on side effects of febuxostat. An evaluation of the cases according to a causality scale like the Naranjo score was not possible as the information given in the case reports was not detailed enough to do so.

#### 2.2.1. Skin Reactions and DRESS Syndrome

The occurrence of hypersensitive skin reactions in conjunction with febuxostat therapy is described for 6 patients. The symptoms described range from itching and erythema [61] to “granulomatous eruption” [62] and “eruptive maculae” [63] to a potentially lethal DRESS syndrome [64,65,66]. DRESS syndrome is a special variant of hypersensitivity reaction (DRESS: drug reaction with eosinophilia and systemic symptoms) [64]. It is described in conjunction with febuxostat therapy in three case reports [64,65,66]. In two of the three patients reported with DRESS syndrome, an intolerance for allopurinol had occurred in the past [65,66]; this event had probably also occurred once in the past with the third patient during allopurinol treatment [64]. Skin reactions occurred in the past during therapy with allopurinol in a total of five of six patients (83.5%) with hypersensitive skin reactions. All described patients recovered. No patient died, see [61,62,63,64,65,66].

#### 2.2.2. Rhabdomyolysis

Three cases of rhabdomyolysis have been described [67,68,69]. One of the patients had a history of a hypersensitive reaction to allopurinol [68]. Two of the patients had impaired renal function with a eGFR of 45 mL/min [67] and 35 ± 7 mL/min [68]. The authors saw a correlation with the development of rhabdomyolysis and the co-administration of a statin together with colchicine [67] or a fibrate [68] parallel to a therapy with febuxostat. In the third case, no conclusions were drawn to the causality of rhabdomyolysis [69]. There is no indication that febuxostat could be the sole cause for the occurrence of a dreaded rhabdomyolysis.

#### 2.2.3. Agranulocytosis

In one paper, two cases of agranulocytosis in the context of treatment with febuxostat are described [70]. Both patients had chronic renal insufficiency [70]. In this context, a correlation with the therapy with febuxostat was neither proven nor excluded [70].

#### 2.2.4. Glomerulonephritis 

Izzedine et al. [71] describe the case of a 63-year-old man who developed acute kidney failure with nephrotic syndrome after five months of therapy with febuxostat at an unknown dosage [71]. An ANCA-positive Pauci-immune glomerulonephritis was diagnosed, the cause of which the authors viewed as the therapy with febuxostat [71].

#### 2.2.5. Acute Liver Disease

Bohm et al. [72] described the case of a 34-year-old patient who developed itching, nausea, abdominal pain, fatigue, and jaundice during febuxostat therapy [72]. The authors suspect the therapy with febuxostat to be the cause for the development of this acute liver disease [72]. The authors made no statements regarding an association with febuxostat or any other causal or predisposing factors.

## 3. Discussion

According to the findings of the present evaluation, febuxostat is well tolerated. There are no major differences between the risk profile for allopurinol and febuxostat in regard to the occurrence of side effects [73]. However, the risk of cardiovascular death was higher under febuxostat therapy compared to allopurinol in patients with gout, whereas the overall mortality was similar [16]. In Seth et al. [74], it was demonstrated that the probability of side effects occurring was 1.12 times higher with allopurinol therapy than with febuxostat [74]. Severe side effects occurred 1.11 times more frequently when allopurinol was administered compared to febuxostat [74]. In another paper, the probability of side effects due to the administration of febuxostat was only 0.94 times as high compared to allopurinol [75]. An effect of febuxostat on arterial blood pressure as an adverse event was not reported. There is indication, that febuxostat lowers blood pressure in patients with normal renal function [76]. An influence on adverse events under febuxostat therapy in the context of the use of alcohol was not reported. With regard to cardiovascular events during febuxostat therapy, further studies need to be conducted to evaluate the effect of febuxostat on cardiovascular events and death.

In the present review of the literature, it was determined that an increase in the occurrence of side effects (without severe side effects) with febuxostat is not to be expected until the daily dose exceeds 120 mg/day. At a dose of 80 and 120 mg of febuxostat per day, the probability of side effects occurring is comparatively lower (relative risk: 0.90 and 0.93) than for therapy with allopurinol (100–300 mg/day) [77]. In the paper by Li et al. [77] it was shown that febuxostat has the lowest risk of side effects compared to allopurinol and other drugs that lower uric acid levels [77].

### 3.1. Renal Function

Based on the available publications, for patients with renal function disorders and extending to those requiring dialysis treatment, therapy is possible at a daily dose of 80 mg of febuxostat without an increase in the occurrence of adverse events.

Allopurinol and its active metabolite oxypurinol are excreted exclusively renally [29]. They can accumulate in patients with renal insufficiency [29]. The excretion of febuxostat is only 50% renal [29]. Its plasma levels (pharmacokinetic) in patients with renal insufficiency up to hemodialysis are stable [29]. In patients with impaired renal function, the allopurinol dose must be reduced [78]. This may result in a decline in the body’s ability to reduce uric acid levels [79]. In addition, renal insufficiency increases the risk of a hypersensitivity reaction to allopurinol [79]. Consequently, a consistent dose reduction is recommended depending on creatinine clearance for allopurinol [79]. According to the findings of the present paper, a dose reduction of febuxostat is not required for patients with impaired renal function or those requiring hemodialysis. Patients with renal insufficiency must be extremely closely monitored under allopurinol therapy with careful dose adjustments. There are always cases in which this is not or not sufficiently practicable.

Bove et al. showed that, in patients with renal dysfunction, the safety profile of febuxostat compared to allopurinol was better regarding relative risk (RR) of adverse events [80]. A study with kidney transplant patients showed that the use of febuxostat in those patients was safe [81]. Using febuxostat, close monitoring of patients with renal insufficiency is not required.

Both allopurinol and febuxostat can be considered in CKD patients. Beside the risk profiles of both drugs in patients with renal insufficiency, cost and availability of intensive monitoring may also help make the decision of which to choose.

### 3.2. Liver Function

The present evaluation revealed an increase in the incidence of adverse events with increasing impairment of liver function. The probability of the occurrence of side effects in patients with pre-existing impairment of liver function is also higher during therapy with allopurinol [82]. This applies specifically to “generalized [...] hypersensitivity reactions” [82]. In patients with impaired liver function, a daily dose of 80 mg febuxostat should not be exceeded according to the findings of the present evaluation. This is consistent with the current product information on febuxostat: “The recommended dose for patients with mild liver function impairment […] is 80 mg” of febuxostat in the treatment of gout [83].

### 3.3. Diabetes Mellitus Type 2

Therapy with febuxostat was tolerated equally well by patients with and without type 2 diabetes mellitus. There was no increase in the incidence of adverse events for patients with or without type 2 diabetes mellitus undergoing therapy with allopurinol [56]. A dose adjustment is required neither for febuxostat nor for allopurinol.

### 3.4. Age Dependence

Patients over 65 years of age developed more adverse events compared to the group of patients under 65 [21,57]. Regarding adverse events, the factors age, morbidity and medication must be considered together. From the findings of the present paper, there is no need for a general dose reduction for older patients [21,57]. It is more important that changes in the side effect profile are monitored for all patients with several secondary disorders and corresponding drug therapy. This is in particular also true for the therapy with allopurinol. Interactions must be watched for specifically in the event of combined therapy of allopurinol with “Mercaptopurine, Azathioprine, Mycophenolatmofetil, cyclosporin, aluminum hydroxide, Theophylline [or] Warfarin” [84]. Patients treated with one of these drugs should not undergo therapy designed to lower uric acid levels with allopurinol, but preferably be treated with febuxostat.

### 3.5. Pre-Existing Allopurinol Intolerance

Patients with a pre-existing allopurinol intolerance are more likely to develop intolerance reactions to febuxostat [28]. Patients with allopurinol intolerance require special medical attention. Patients who exhibit symptoms of a hypersensitivity reaction during therapy with febuxostat should end therapy immediately [28,61,62,64,65,66]. Nevertheless, “hypersensitivity to allopurinol is not a contraindication to febuxostat” [85].

### 3.6. Combination of Febuxostat with NSAIDs 

Combining febuxostat with NSAID does not result in a significant increase in adverse events based on the present evaluation [52]. This combination is an alternative to the combination of allopurinol with an NSAID with regard to side effects.

### 3.7. Combination of Febuxostat with Hydrochlorothiazide

It was shown that the combined therapy with febuxostat and hydrochlorothiazide is well tolerated without a dose adjustment [12]. In a therapy with allopurinol, a simultaneous therapy with a thiazide escalates the risk of a hypersensitivity reaction [86]. Based on the publications analyzed, a therapy with febuxostat to reduce uric acid levels should be introduced for patients treated with hydrochlorothiazide. No adjustment of the febuxostat dose is necessary.

### 3.8. Combination of Febuxostat with Uricosuric Drugs

No severe side effects occurred during the combined therapy of febuxostat together with Lesinurad or Arhalofenat [58,59]. The question of side effects during the co-administration of Lesinurad and allopurinol is the subject of current research (see [87,88]). Before a final assessment of the tolerability of febuxostat in combination with Lesinurad or Arhalofenat can be made, the findings of additional studies must be waited for. Whether the combination of these new uricosuric agents with one of the available xanthine oxidase inhibitors has advantages for the patient cannot be inferred from the available literature.

### 3.9. Combination of Febuxostat with Azathioprine

In two publications [55,60], the authors report pancytopenia and eosinophilia when febuxostat and azathioprine are administered together which they consider a possible interaction of the two substances. In the scope of allopurinol therapy, the interaction with azathioprine is a known complication [89]. Severe side effects can occur [90]. To reduce this risk, the allopurinol dose should be reduced when combined with azathioprine. In addition, within the scope of allopurinol therapy, an increased risk of bone marrow suppression with a potential agranulocytosis is known through drug interaction with azathioprine [84]. Consequently, these complications and interactions should also be considered in febuxostat therapy if corresponding symptoms occur and immediate action taken, that is, the patient should discontinue taking the drug. Given an existing therapy with azathioprine, a therapy designed to lower uric acid levels, preferably with febuxostat, should follow.

### 3.10. Skin Reactions and DRESS Syndrome

In the present analysis, a pre-existing allopurinol intolerance was identified as a risk factor for the development of an intolerance to febuxostat [61,62,64,65,66]. Under therapy with allopurinol, up to 0.4% develop a DRESS syndrome [78]. For allopurinol, the greatest risk factor for the occurrence of a hypersensitivity reaction is an impairment in renal function [78]. Another risk factor is therapy of asymptomatic hyperuricemia with allopurinol [78]. A comparable correlation was not described for febuxostat.

### 3.11. Rhabdomyolysis

In the paper by Kang et al. [67], the authors hypothesize that the combination of febuxostat, colchicine, and rosuvastatin may have led to an increase in the plasma levels of one or more of these drugs and may thus have caused rhabdomyolysis [67]. The risk of developing rhabdomyolysis following co-administration of colchicine together with a statin is described in various case histories ([91,92,93,94,95,96]). The simultaneous existence of impaired renal function also raises the risk for rhabdomyolysis under such combination therapy [91,92,93,96]. The occurrence of rhabdomyolysis is also described in the combination of colchicine with fibrates [97]. With regard to patient safety, the symptoms of this side effect must expressly be watched for, and the patient must be informed if—in addition to febuxostat—a therapy with a statin, colchicine or a fibrate is intended. In this situation, regular control of the creatine kinase is to be recommended to be able to catch rhabdomyolysis at an early state. Liu et al. presented a study with 1332 patients with chronic kidney disease [98]. Forty-one patients developed a myopathy [98]. “Febuxostat was the culprit agent […] in only two patients […]” [98]. Further studies are required to identify the pathophysiologic relation between febuxostat and myopathy.

### 3.12. Agranulocytosis

In the paper by Kobayashi et al. [70], a correlation with febuxostat therapy could neither be proven nor excluded. Consequently, it is not possible to definitively state whether febuxostat can cause agranulocytosis based on the present evaluation. Further studies must be waited for.

### 3.13. Glomerulonephritis

One case of an ANCA-positive Pauci-immune glomerulonephritis has been published in which the authors see the cause in therapy with febuxostat [71]. The exact pathophysiological correlation remains unclarified.

### 3.14. Acute Liver Disease

A connection between therapy with febuxostat and an underlying acute liver disease is suspected [72]. The present evaluation has shown that changes in liver values are also one of the side effects of therapy with febuxostat. This side effect was also the main reason reported for participants dropping out of the studies prematurely considered in the present review (see studies in Table A1).

The risk of liver damage due to allopurinol is known. In a paper by Singer and Wallace, 72 patients are cited as having developed a hypersensitivity reaction to allopurinol [99]. Acute liver cell damage was diagnosed in 37 patients (51.3%) [99].

In the event of corresponding symptoms or changes in laboratory values during therapy with febuxostat, the prescribing physician should consider the risk of an acute liver disease and closely monitor therapy control.

## 4. Materials and Methods

Publications on the side effects and/or interactions of febuxostat were sought in the databases PubMed and Google scholar (last query on 20 March 2018) as well as in the list of references in located papers. The search terms used were: febuxostat, adverse effects, adverse events, side effects, interaction, safety. Original clinical studies, concerned with the treatment of patients with hyperuricemia and gout with febuxostat, were considered for this review in the case of presenting the number of patients with adverse events. Forty-eight original papers with 12,323 patients, six secondary analyses in these papers, as well as 14 case reports of 15 patients were systematically evaluated. The papers were evaluated for events in conjunction with therapy with febuxostat. These included side effects, severe side effects, dropping out of the study prematurely, as well as the death of a study participant. The total number of side effects that occurred is the sum of mild side effects (referred to as “side effect”) and severe side effects (referred to as “severe side effect”).

The rating by mild (e.g., headache, diarrhea, nausea) and severe side effect (e.g., hypersensitivity reactions, major changes in liver values, cardiovascular events) is based on the authors’ rating in their respective publication. The classification is not based on a standard definition.

Events which were not evaluated as being connected to febuxostat in the respective publications were not included in this paper’s evaluation. The comparison to allopurinol was made based on reviews addressing the side effects or interactions of allopurinol.

## 5. Conclusions

In the interest of preventing hypersensitivity reactions, such as skin reactions or DRESS syndrome, allopurinol should be carefully used and the therapy should be closely monitored in patients with impairment of renal or liver function. Febuxostat should be considered for therapy. Higher CV death rates during febuxostat therapy must also be taken into consideration, although there is no difference in the overall death rate between patients being treated with febuxostat and those with allopurinol. No across-the-board dose adjustment for febuxostat is needed for older patients. Existing concomitant medications must be considered in all age groups. Therapy with allopurinol is contraindicated for patients with a previously known intolerance for allopurinol. In these cases, febuxostat can still be used to treat hyperuricemia and gout whereby the risk of intolerances is elevated. This must be watched in daily clinical practice. In the majority of the published case reports, no correlation of side effects was shown to therapy with febuxostat.

The present analysis has shown that febuxostat is well tolerated and a modern and safe alternative to allopurinol therapy. It can be expected that these findings indicate that the agent febuxostat will continue to become more important in daily clinical practice. This will also have a positive impact on the quality of care and therapeutic safety of patients with hyperuricemia and gout.

## Figures and Tables

**Table 1:** Adverse events in patients depending on liver function (number of patients examined per group (column 1); liver function of patient group (column 2); patients with at least one side effect (column 3); patients with at least one severe side effect (column 4); publications reviewed (column 5); differentiation between side effect and severe side effect in accordance with the publications, no standard definition).

Number of Patients with Febuxostat Therapy	Liver Function	Number of Patients with at Least One Side Effect (Share in %)	Number of Patients with at Least One Severe Side Effect (Share in %)	Source
**12**	Normal	3 (25.0)	0	[50]
**8**	Mildly limited	5 (63.0)	0	
**8**	Moderately limited	6 (75.0)	0	

**Table 2:** Side effects during therapy with febuxostat and NSAID (applied NSAID (column 1); number of patients who were administered the NSAID (column 2); patients with at least one side effect during therapy with febuxostat (column 3), febuxostat together with NSAID (column 4); NSAID (column 5); publications reviewed (column 6)).

NSAID	Number of Patients Examined	Number of Patients with at Least One Side Effect (Share in % a Study Population)			Source
		Febuxostat	Febuxostat + NSAID	NSAID	
**Indometacin**	27	2 (8.0)	6 (22.0)	7 (27.0)	[52]
**Naproxen**	26	7 (28.0)	9 (35.0)	7 (26.0)	

## Appendix A

**Table A1:** Adverse events in the original works (number of patients examined per study (column 1); patients with at least one side effect (column 2); patients with at least one severe side effect (column 3); patients who dropped out of the study prematurely (column 4); patients who died (column 5); publications reviewed (column 6); differentiation between side effect and severe side effect according to the respective publication, no standard definition).

Number of Patients with Febuxostat Therapy	Number of Patients with at Least One Side Effect (Share in %)	Number of Patients with at Least One Severe Side Effect (Share in %)	Number of Patients to Drop out Prematurely (Share in %)	Number of Patients Who Died (Share in %)	Source
**154**	60 (38.9)	0	9 (5.8)	0	[26]
**15**	1 (6.7)	0	0	0	[23]
**507**	123 (24.3)	32 (6.3)	39 (7.7)	0	[8]
**115**	14 (12.2)	3 (2.6)	5 (4.3)	0	[49]
**32**	10 (31.3)	0	0	0	[22]
**28**	14 (50.0)	0	0	0	[50]
**118**	57 (48.3)	0	10 (8.5)	0	[51]
**51**	9 (17.6)	0	0	0	[52]
**92**	26 (28.3)	2 (2.2)	3 (3.3)	0	[20]
**48**	21 (43.8)	0	0	0	[21]
**670**	462 (69.0)	25 (3.7)	61 (9.1)	0	[9]
**116**	106 (91.0)	0	13 (11.2)	0	[10]
**1513**	839 (55.5)	47 (3.1)	110 (7.3)	0	[11]
**34**	14 (41.2)	0	0	0	[12]
**13**	1 (7.7)	1 (7.7)	0	0	[32]
**6**	3 (50.0)	0	0	0	[18]
**29**	3 (10.3)	0	0	0	[17]
**20**	8 (40.0)	0	0	0	[45]
**122**	10 (8.2)	0	0	0	[46]
**171**	63 (36.8)	0	6 (3.5)	0	[47]
**161**	39 (24.2)	1 (0.6)	0	0	[48]
**69**	11 (15.9)	0	1 (1.5)	0	[13]
**39**	12 (30.8)	0	0	0	[25]
**12**	2 (16.7)	0	0	0	[24]
**33**	20 (60.6)	0	2 (6.1)	0	[36]
**17**	5 (29.4)	0	0	0	[37]
**100**	1 (1.0)	0	2 (2.0)	0	[27]
**344**	113 (32.8)	0	21 (6.1)	0	[41]
**38**	1 (2.6)	0	0	0	[53]
**82**	10 (12.2)	0	0	0	[33]
**70**	5 (7.1)	0	4 (5.7) (included in pat. with ≥ 1 side effect)	0	[14]
**26**	0	0	0	0	[43]
**5948**	152 (2.6)	15 (0.25)	36 (0.6)	0	[2]
**22**	0	1 (4.5)	0	0	[42]
**151**	6 (4.0)	0	0	0	[34]
**36**	0	0	0	0	[19]
**51**	5 (9.8)	0	0	0	[29]
**45**	2 (4.4)	0	0	0	[38]
**336**	91 (27.1)	1 (0.3)	6 (1.8)	0	[35]
**89**	33 (37.1)	0	0	0	[30]
**101**	4 (4.0)	0	0	0	[28]
**160**	27 (16.9)	0	5 (3.1)	0	[39]
**294**	18 (6.1)	0	7 (2.4)	0	[44]
**83**	0	0	6 (7.2)	0	[40]
**64**	53 (82.8)	13 (20.3)	8 (12.5)	1 (1.6)	[15]
**54**	8 (14.8)	2 (3.7)	0	0	[31]
**12,279**	2462 (20.1)	143 (1.2)	354 (2.9)	1 (0.008)	TOTAL

## Appendix B

**Table A2:** Adverse events in the secondary analyses (number of patients examined per study (column 1); patients with at least one side effect (column 2); patients with at least one severe side effect (column 3); patients who dropped out of the study prematurely (column 4); patients who died (column 5); publications reviewed (column 6); differentiation between side effect and severe side effect according to the respective publication, no standard definition).

Number of Patients with Febuxostat Therapy	Number of Patients with at Least One Side Effect (Share in %)	Number of Patients with at Least One Severe Side Effect (Share in %)	Number of Patients to Drop Out Prematurely (Share in %)	Number of Patients Who Died (Share in %)	Source
**1513**	ND	49 (3.2)	ND	2 (0.7)	[57]
**116**	106 (91.0)	21 (18.0)	13 (11.2)	0	[100]
**137**	100 (71.9)	9 (6.5)	ND	1 (0.7)	[101]
**243**	145 (59.7)	17 (7.0)	34 (14.0)	0	[102]
**1399**	770 (55.0)	44 (3.2)	0	0	[103]
**1462**	812 (55.5)	47 (3.2)	ND	0	[56]
**4870**	1933 (39.7)	187 (3.8)	47 (1.0)	3 (0.06)	Total

## References

[1] Tausche A.-K., Jansen T.L., Schroder H.-E., Bornstein S.R., Aringer M., Muller-Ladner U. (2009). Gout—Current diagnosis and treatment. Deutsches Ärzteblatt Int..

[2] Tausche A.-K., Reuss-Borst M., Koch U. (2014). Urate lowering therapy with febuxostat in daily practice—A multicentre, open-label, prospective observational study. Int. J. Rheumatol..

[3] Edwards N.L. (2009). Febuxostat: A new treatment for hyperuricaemia in gout. Rheumatology.

[4] Spiekermann S. (2003). Electron spin resonance characterization of vascular xanthine and NAD(P)H oxidase activity in patients with coronary artery disease: Relation to endothelium-dependent vasodilation. Circulation.

[5] White C.R., Darley-Usmar V., Berrington W.R., McAdams M., Gore J.Z., Thompson J.A., Parks D.A., Tarpey M.M., Freeman B.A. (1996). Circulating plasma xanthine oxidase contributes to vascular dysfunction in hypercholesterolemic rabbits. Proc. Natl. Acad. Sci. USA.

[6] Cardillo C., Kilcoyne C.M., Cannon R.O., Quyyumi A.A., Panza J.A. (1997). Xanthine oxidase inhibition with oxypurinol improves endothelial vasodilator function in hypercholesterolemic but not in hypertensive patients. Hypertension.

[7] Tausche A.-K., Christoph M., Forkmann M., Richter U., Kopprasch S., Bielitz C., Aringer M., Wunderlich C. (2014). As compared to allopurinol, urate-lowering therapy with febuxostat has superior effects on oxidative stress and pulse wave velocity in patients with severe chronic tophaceous gout. Rheumatol. Int..

[8] Becker M.A., Schumacher H.R., Wortmann R.L., MacDonald P.A., Eustace D., Palo W.A., Streit J., Joseph-Ridge N. (2005). Febuxostat compared with allopurinol in patients with hyperuricemia and gout. N. Engl. J. Med..

[9] Schumacher H.R., Becker M.A., Wortmann R.L., MacDonald P.A., Hunt B., Streit J., Lademacher C., Joseph-Ridge N. (2008). Effects of febuxostat versus allopurinol and placebo in reducing serum urate in subjects with hyperuricemia and gout: A 28-week, phase III, randomized, double-blind, parallel-group trial. Arthritis Rheum..

[10] Schumacher H.R., Becker M.A., Lloyd E., MacDonald P.A., Lademacher C. (2009). Febuxostat in the treatment of gout: 5-yr findings of the FOCUS efficacy and safety study. Rheumatology.

[11] Becker M.A., Schumacher H.R., Espinoza L.R., Wells A.F., MacDonald P., Lloyd E., Lademacher C. (2010). The urate-lowering efficacy and safety of febuxostat in the treatment of the hyperuricemia of gout: The CONFIRMS trial. Arthritis Res. Ther..

[12] Grabowski B., Khosravan R., Wu J.-T., Vernillet L., Lademacher C. (2010). Effect of hydrochlorothiazide on the pharmacokinetics and pharmacodynamics of febuxostat, a non-purine selective inhibitor of xanthine oxidase. Br. J. Clin. Pharmacol..

[13] Kamatani N., Fujimori S., Hada T., Hosoya T., Kohri K., Nakamura T., Ueda T., Yamamoto T., Yamanaka H., Matsuzawa Y. (2011). Placebo-controlled, double-blind study of the non-purine-selective xanthine oxidase inhibitor Febuxostat (TMX-67) in patients with hyperuricemia including those with gout in Japan: Phase 3 clinical study. J. Clin. Rheumatol..

[14] Shibagaki Y., Ohno I., Hosoya T., Kimura K. (2014). Safety, efficacy and renal effect of febuxostat in patients with moderate-to-severe kidney dysfunction. Hypertens. Res. Off. J. Jpn. Soc. Hypertens..

[15] Saag K.G., Whelton A., Becker M.A., MacDonald P., Hunt B., Gunawardhana L. (2016). Impact of febuxostat on renal function in gout patients with moderate-to-severe renal impairment. Arthritis Rheumatol..

[16] White W.B., Saag K.G., Becker M.A., Borer J.S., Gorelick P.B., Whelton A., Hunt B., Castillo M. (2018). Cardiovascular Safety of Febuxostat or Allopurinol in Patients with Gout. N. Engl. J. Med..

[17] Hosoya T., Ohno I. (2011). A repeated oral administration study of febuxostat (TMX-67), a non-purine-selective inhibitor of xanthine oxidase, in patients with impaired renal function in Japan: Pharmacokinetic and pharmacodynamic study. J. Clin. Rheumatol..

[18] Grabowski B.A., Khosravan R., Vernillet L., Mulford D.J. (2011). Metabolism and excretion of 14C febuxostat, a novel nonpurine selective inhibitor of xanthine oxidase, in healthy male subjects. J. Clin. Pharmacol..

[19] Zhang M., Di X., Xu L., Xu J., Yang Y., Jiang N., Song L., Xu X. (2014). Pharmacokinetics and pharmacodynamics of febuxostat under fasting conditions in healthy individuals. Exp. Ther. Med..

[20] Khosravan R., Grabowski B., Wu J.-T., Joseph-Ridge N., Vernillet L. (2008). Effect of food or antacid on pharmacokinetics and pharmacodynamics of febuxostat in healthy subjects. Br. J. Clin. Pharmacol..

[21] Khosravan R., Kukulka M.J., Wu J.-T., Joseph-Ridge N., Vernillet L. (2008). The effect of age and gender on pharmacokinetics, pharmacodynamics, and safety of febuxostat, a novel nonpurine selective inhibitor of xanthine oxidase. J. Clin. Pharmacol..

[22] Mayer M.D., Khosravan R., Vernillet L., Wu J.-T., Joseph-Ridge N., Mulford D.J. (2005). Pharmacokinetics and pharmacodynamics of febuxostat, a new non-purine selective inhibitor of xanthine oxidase in subjects with renal impairment. Am. J. Ther..

[23] Hoshide S., Takahashi Y., Ishikawa T., Kubo J., Tsuchimoto M., Komoriya K., Ohno I., Hosoya T. (2004). PK/PD and safety of a single dose of TMX-67 (febuxostat) in subjects with mild and moderate renal impairment. Nucleosides Nucleotides Nucleic Acids.

[24] Tsai M., Wu J.-T., Gunawardhana L., Naik H. (2012). The effects of xanthine oxidase inhibition by febuxostat on the pharmacokinetics of theophylline. Int. J. Clin. Pharmacol. Ther..

[25] Naik H., Wu J.-T., Palmer R., McLean L. (2012). The effects of febuxostat on the pharmacokinetic parameters of rosiglitazone, a CYP2C8 substrate. Br. J. Clin. Pharmacol..

[26] Becker M.A., Kisicki J., Khosravan R., Wu J., Mulford D., Hunt B., MacDonald P., Joseph-Ridge N. (2004). Febuxostat (TMX-67), a novel, non-purine, selective inhibitor of xanthine oxidase, is safe and decreases serum urate in healthy volunteers. Nucleosides Nucleotides Nucleic Acids.

[27] Hiramitsu S., Ishiguro Y., Matsuyama H., Yamada K., Kato K., Noba M., Uemura A., Matsubara Y., Yoshida S., Kani A. (2014). Febuxostat (Feburic tablet) in the management of hyperuricemia in a general practice cohort of Japanese patients with a high prevalence of cardiovascular problems. Clin. Exp. Hypertens..

[28] Bardin T., Chales G., Pascart T., Flipo R.-M., Korng Ea H., Roujeau J.-C., Delayen A., Clerson P. (2016). Risk of cutaneous adverse events with febuxostat treatment in patients with skin reaction to allopurinol. A retrospective, hospital-based study of 101 patients with consecutive allopurinol and febuxostat treatment. Jt. Bone Spine.

[29] Hira D., Chisaki Y., Noda S., Araki H., Uzu T., Maegawa H., Yano Y., Morita S.-Y., Terada T. (2015). Population pharmacokinetics and therapeutic efficacy of febuxostat in patients with severe renal impairment. Pharmacology.

[30] Yamamoto T., Hidaka Y., Inaba M., Ishimura E., Ooyama H., Kakuta H., Moriwaki Y., Higami K., Ohtawara A., Hosoya T. (2015). Effects of febuxostat on serum urate level in Japanese hyperuricemia patients. Mod. Rheumatol..

[31] Yu K.-H., Lai J.-H., Hsu P.-N., Chen D.-Y., Chen C.-J., Lin H.-Y. (2016). Safety and efficacy of oral febuxostat for treatment of HLA-B*5801-negative gout: A randomized, open-label, multicentre, allopurinol-controlled study. Scand. J. Rheumatol..

[32] Chohan S. (2011). Safety and efficacy of febuxostat treatment in subjects with gout and severe allopurinol adverse reactions. J. Rheumatol..

[33] Mizuno T., Hayashi T., Hikosaka S., Shimabukuro Y., Murase M., Takahashi K., Hayashi H., Yuzawa Y., Nagamatsu T., Yamada S. (2014). Efficacy and safety of febuxostat in elderly female patients. Clin. Interv. Aging.

[34] Wang Y.S., Ng S.P., Kuo L.H., Chien S.Y. (2014). The effectiveness and safety of febuxostat: An experience in medical center in Taiwan. Value Health.

[35] Xu S., Liu X., Ming J., Chen S., Wang Y., Liu X., Liu H., Peng Y., Wang J., Lin J. (2015). A phase 3, multicenter, randomized, allopurinol-controlled study assessing the safety and efficacy of oral febuxostat in Chinese gout patients with hyperuricemia. Int. J. Rheum. Dis..

[36] Goldfarb D.S., MacDonald P.A., Gunawardhana L., Chefo S., McLean L. (2013). Randomized controlled trial of febuxostat versus allopurinol or placebo in individuals with higher urinary uric acid excretion and calcium stones. Clin. J. Am. Soc. Nephrol..

[37] Akimoto T., Morishita Y., Ito C., Iimura O., Tsunematsu S., Watanabe Y., Kusano E., Nagata D. (2014). Febuxostat for hyperuricemia in patients with advanced chronic kidney disease. Drug Target Insights.

[38] Sircar D., Chatterjee S., Waikhom R., Golay V., Raychaudhury A., Chatterjee S., Pandey R. (2015). Efficacy of febuxostat for slowing the GFR decline in patients with CKD and asymptomatic hyperuricemia: A 6-month, double-blind, randomized, placebo-controlled trial. Am. J. Kidney Dis..

[39] Ito H., Antoku S., Abe M., Omoto T., Shinozaki M., Nishio S., Mifune M., Togane M., Nakata M., Yamashita T. (2016). Comparison of the renoprotective effect of febuxostat for the treatment of hyperuricemia between patients with and without type 2 diabetes mellitus: A retrospective observational study. Intern. Med..

[40] Quilis N., Andres M., Gil S., Ranieri L., Vela P., Pascual E. (2016). Febuxostat for patients with gout and severe chronic kidney disease: Which is the appropriate dosage? Comment on the article by Saag et al.. Arthritis Rheumatol..

[41] Huang X., Du H., Gu J., Zhao D., Jiang L., Li X., Zuo X., Liu Y., Li Z., Li X. (2014). An allopurinol-controlled, multicenter, randomized, double-blind, parallel between-group, comparative study of febuxostat in Chinese patients with gout and hyperuricemia. Int. J. Rheum. Dis..

[42] Tojimbara T., Nakajima I., Yashima J., Fuchinoue S., Teraoka S. (2014). Efficacy and safety of febuxostat, a novel nonpurine selective inhibitor of xanthine oxidase for the treatment of hyperuricemia in kidney transplant recipients. Transplant. Proc..

[43] Sofue T., Inui M., Hara T., Nishijima Y., Moriwaki K., Hayashida Y., Ueda N., Nishiyama A., Kakehi Y., Kohno M. (2014). Efficacy and safety of febuxostat in the treatment of hyperuricemia in stable kidney transplant recipients. Drug Des. Dev. Ther..

[44] Lim D.-H., Oh J.S., Ahn S.M., Hong S., Kim Y.-G., Lee C.-K., Choi S.W., Yoo B. (2016). Febuxostat in hyperuricemic patients with advanced CKD. Am. J. Kidney Dis..

[45] Kamatani N., Fujimori S., Hada T., Hosoya T., Kohri K., Nakamura T., Ueda T., Yamamoto T., Yamanaka H., Matsuzawa Y. (2011). An allopurinol-controlled, multicenter, randomized, open-label, parallel between-group, comparative study of febuxostat (TMX-67), a non-purine-selective inhibitor of xanthine oxidase, in patients with hyperuricemia including those with gout in Japan: Phase 2 exploratory clinical study. J. Clin. Rheumatol..

[46] Kamatani N., Fujimori S., Hada T., Hosoya T., Kohri K., Nakamura T., Ueda T., Yamamoto T., Yamanaka H., Matsuzawa Y. (2011). An allopurinol-controlled, randomized, double-dummy, double-blind, parallel between-group, comparative study of febuxostat (TMX-67), a non-purine-selective inhibitor of xanthine oxidase, in patients with hyperuricemia including those with gout in Japan: Phase 3 clinical study. J. Clin. Rheumatol..

[47] Kamatani N., Fujimori S., Hada T., Hosoya T., Kohri K., Nakamura T., Ueda T., Yamamoto T., Yamanaka H., Matsuzawa Y. (2011). Multicenter, open-label study of long-term administration of febuxostat (TMX-67) in Japanese patients with hyperuricemia including gout. J. Clin. Rheumatol..

[48] Kamatani N., Fujimori S., Hada T., Hosoya T., Kohri K., Nakamura T., Ueda T., Yamamoto T., Yamanaka H., Matsuzawa Y. (2011). Placebo-controlled double-blind dose-response study of the non-purine-selective xanthine oxidase inhibitor febuxostat (TMX-67) in patients with hyperuricemia (including gout patients) in japan: Late phase 2 clinical study. J. Clin. Rheumatol..

[49] Becker M.A., Schumacher H.R., Wortmann R.L., MacDonald P.A., Palo W.A., Eustace D., Vernillet L., Joseph-Ridge N. (2005). Febuxostat, a novel nonpurine selective inhibitor of xanthine oxidase: A twenty-eight-day, multicenter, phase II, randomized, double-blind, placebo-controlled, dose-response clinical trial examining safety and efficacy in patients with gout. Arthritis Rheum..

[50] Khosravan R., Grabowski B.A., Mayer M.D., Wu J.-T., Joseph-Ridge N., Vernillet L. (2006). The effect of mild and moderate hepatic impairment on pharmacokinetics, pharmacodynamics, and safety of febuxostat, a novel nonpurine selective inhibitor of xanthine oxidase. J. Clin. Pharmacol..

[51] Khosravan R., Grabowski B.A., Wu J.-T., Joseph-Ridge N., Vernillet L. (2006). Pharmacokinetics, pharmacodynamics and safety of febuxostat, a non-purine selective inhibitor of xanthine oxidase, in a dose escalation study in healthy subjects. Clin. Pharmacokinet..

[52] Khosravan R., Wu J.-T., Joseph-Ridge N., Vernillet L. (2006). Pharmacokinetic interactions of concomitant administration of febuxostat and NSAIDs. J. Clin. Pharmacol..

[53] Maie K., Yokoyama Y., Kurita N., Minohara H., Yanagimoto S., Hasegawa Y., Homma M., Chiba S. (2014). Hypouricemic effect and safety of febuxostat used for prevention of tumor lysis syndrome. SpringerPlus.

[54] Becker M.A., Schumacher H.R., MacDonald P.A., Lloyd E., Lademacher C. (2009). Clinical efficacy and safety of successful longterm urate lowering with febuxostat or allopurinol in subjects with gout. J. Rheumatol..

[55] Kaczmorski S., Doares W., Winfrey S., Al-Geizawi S., Farney A., Rogers J., Stratta R. (2011). Gout and transplantation: New treatment option–Same old drug interaction. Transplantation.

[56] Becker M.A., MacDonald P.A., Hunt B.J., Jackson R.L. (2013). Diabetes and gout: Efficacy and safety of febuxostat and allopurinol. Diabetes Obes. Metab..

[57] Becker M.A., MacDonald P.A., Hunt B., Gunawardhana L. (2011). Treating hyperuricemia of gout: Safety and efficacy of febuxostat and allopurinol in older versus younger subjects. Nucleosides Nucleotides Nucleic Acids.

[58] Fleischmann R., Kerr B., Yeh L.-T., Suster M., Shen Z., Polvent E., Hingorani V., Quart B., Manhard K., Miner J.N. (2014). Pharmacodynamic, pharmacokinetic and tolerability evaluation of concomitant administration of lesinurad and febuxostat in gout patients with hyperuricaemia. Rheumatology.

[59] Steinberg A.S., Vince B.D., Choi Y.-J., Martin R.L., McWherter C.A., Boudes P.F. (2016). The pharmacodynamics, pharmacokinetics, and safety of arhalofenate in combination with febuxostat when treating hyperuricemia associated with gout. J. Rheumatol..

[60] Dore M., Frenette A.J., Mansour A.-M., Troyanov Y., Begin J. (2014). Febuxostat as a novel option to optimize thiopurines’ metabolism in patients with inadequate metabolite levels. Ann. Pharmacother..

[61] Abeles A.M. (2012). Febuxostat hypersensitivity. J. Rheumatol..

[62] Laura A., Luca P., Luisa P.A. (2014). Interstitial granulomatous drug reaction due to febuxostat. Indian J. Dermatol. Venereol. Leprol..

[63] Oda T., Sawada Y., Ohmori S., Omoto D., Haruyama S., Yoshioka M., Nishio D., Nakamura M. (2016). Fixed drug eruption-like macules caused by febuxostat. Eur. J. Dermatol..

[64] Chou H.-Y., Chen C.-B., Cheng C.-Y., Chen Y.-A., Ng C.Y., Kuo K.-L., Chen W.-L., Chen C.-H. (2015). Febuxostat-associated drug reaction with eosinophilia and systemic symptoms (DRESS). J. Clin. Pharm. Ther..

[65] Paschou E., Gavriilaki E., Papaioannou G., Tsompanakou A., Kalaitzoglou A., Sabanis N. (2016). Febuxostat hypersensitivity: Another cause of DRESS syndrome in chronic kidney disease?. Eur. Ann. Allergy Clin. Immunol..

[66] Lien Y.-H.H., Logan J.L. (2017). Cross-reactions between allopurinol and febuxostat. Am. J. Med..

[67] Kang Y., Kim M.J., Jang H.N., Bae E.J., Yun S., Cho H.S., Chang S.-H., Park D.J. (2014). Rhabdomyolysis associated with initiation of febuxostat therapy for hyperuricaemia in a patient with chronic kidney disease. J. Clin. Pharm. Ther..

[68] Ghosh D., McGann P.M., Furlong T.J., Day R.O. (2015). Febuxostat-associated rhabdomyolysis in chronic renal failure. Med. J. Aust..

[69] Chahine G., Saleh K., Ghorra C., Khoury N., Khalife N., Fayad F. (2016). Febuxostat-associated eosinophilic polymyositis in marginal zone lymphoma. Jt. Bone Spine.

[70] Kobayashi S., Ogura M., Hosoya T. (2013). Acute neutropenia associated with initiation of febuxostat therapy for hyperuricaemia in patients with chronic kidney disease. J. Clin. Pharm. Ther..

[71] Izzedine H., Boulanger H., Gueutin V., Rouvier P., Deray G. (2012). ANCA-positive pauci-immune glomerulonephritis and febuxostat treatment. Clin. Kidney J..

[72] Bohm M., Vuppalanchi R., Chalasani N. (2016). Febuxostat-induced acute liver injury. Hepatology.

[73] Castrejon I., Toledano E., Rosario M.P., Loza E., Perez-Ruiz F., Carmona L. (2015). Safety of allopurinol compared with other urate-lowering drugs in patients with gout: A systematic review and meta-analysis. Rheumatol. Int..

[74] Seth R., Kydd A.S.R., Buchbinder R., Bombardier C., Edwards C.J. (2014). Allopurinol for chronic gout. Cochrane Database Syst. Rev..

[75] Faruque L.I., Ehteshami-Afshar A., Wiebe N., Tjosvold L., Homik J., Tonelli M. (2013). A systematic review and meta-analysis on the safety and efficacy of febuxostat versus allopurinol in chronic gout. Semin. Arthritis Rheum..

[76] Gunawardhana L., McLean L., Punzi H.A., Hunt B., Palmer R.N., Whelton A., Feig D.I. (2017). Effect of Febuxostat on Ambulatory Blood Pressure in Subjects With Hyperuricemia and Hypertension: A Phase 2 Randomized Placebo-Controlled Study. J. Am. Heart Assoc..

[77] Li S., Yang H., Guo Y., Wei F., Yang X., Li D., Li M., Xu W., Li W., Sun L. (2016). Comparative efficacy and safety of urate-lowering therapy for the treatment of hyperuricemia: A systematic review and network meta-analysis. Sci. Rep..

[78] Markel A. (2005). Allopurinol-induced DRESS syndrome. Isr. Med. Assoc. J..

[79] Dalbeth N., Stamp L. (2007). Allopurinol dosing in renal impairment: Walking the tightrope between adequate urate lowering and adverse events. Semin. Dial..

[80] Bove M., Cicero A.F.G., Veronesi M., Borghi C. (2017). An evidence-based review on urate-lowering treatments: Implications for optimal treatment of chronic hyperuricemia. Vasc. Health Risk Manag..

[81] Baek C.H., Kim H., Yang W.S., Han D.J., Park S.-K. (2017). Efficacy and Safety of Febuxostat in Kidney Transplant Patients. Exp. Clin. Transplant..

[82] Ratiopharm GmbH (2016). Fachinformation zu “Allopurinol-ratiopharm 100/300 mg Tabletten”. http://www.ratiopharm.de/index.php?eID=dumpFile&t=f&f=70732&g=-1&r=1894%2C1894&token=38cd525615b9b72e43152a6306fbb025793a5c41.

[83] Berlin-Chemie, A.G. (2017). Fachinformation Adenuric. http://www.fachinfo.de/pdf/012335.

[84] Pea F. (2005). Pharmacology of drugs for hyperuricemia. Mechanisms, kinetics and interactions. Contrib. Nephrol..

[85] Waller A., Jordan K.M. (2017). Use of febuxostat in the management of gout in the United Kingdom. Ther. Adv. Musculoskelet. Dis..

[86] Chao J., Terkeltaub R. (2009). A critical reappraisal of allopurinol dosing, safety, and efficacy for hyperuricemia in gout. Curr. Rheumatol. Rep..

[87] Perez-Ruiz F., Sundy J.S., Miner J.N., Cravets M., Storgard C. (2016). Lesinurad in combination with allopurinol: Results of a phase 2, randomised, double-blind study in patients with gout with an inadequate response to allopurinol. Ann. Rheum. Dis..

[88] Bardin T., Keenan R.T., Khanna P.P., Kopicko J., Fung M., Bhakta N., Adler S., Storgard C., Baumgartner S., So A. (2016). Lesinurad in combination with allopurinol: A randomised, double-blind, placebo-controlled study in patients with gout with inadequate response to standard of care (the multinational CLEAR 2 study). Ann. Rheum. Dis..

[89] Stamp L.K., Jordan S. (2011). The challenges of gout management in the elderly. Drugs Aging.

[90] Gearry R.B., Day A.S., Barclay M.L., Leong R.W.L., Sparrow M.P. (2010). Azathioprine and allopurinol: A two-edged interaction. J. Gastroenterol. Hepatol..

[91] Hsu W.-C., Chen W.-H., Chang M.-T., Chiu H.-C. (2002). Colchicine-induced acute myopathy in a patient with concomitant use of simvastatin. Clin. Neuropharmacol..

[92] Baker S.K., Goodwin S., Sur M., Tarnopolsky M.A. (2004). Cytoskeletal myotoxicity from simvastatin and colchicine. Muscle Nerve.

[93] Alayli G., Cengiz K., Canturk F., Durmus D., Akyol Y., Menekse E.B. (2005). Acute myopathy in a patient with concomitant use of pravastatin and colchicine. Ann. Pharmacother..

[94] Atasoyu E.M., Evrenkaya T.R., Solmazgul E. (2005). Possible colchicine rhabdomyolysis in a fluvastatin-treated patient. Ann. Pharmacother..

[95] Tufan A., Dede D.S., Cavus S., Altintas N.D., Iskit A.B., Topeli A. (2006). Rhabdomyolysis in a patient treated with colchicine and atorvastatin. Ann. Pharmacother..

[96] Sarullo F.M., Americo L., Di Franco A., Di Pasquale P. (2010). Rhabdomyolysis induced by co-administration of fluvastatin and colchicine. Monaldi Arch. Chest Dis..

[97] Leung Y.Y., Yao Hui L.L., Kraus V.B. (2015). Colchicine—Update on mechanisms of action and therapeutic uses. Semin. Arthritis Rheum..

[98] Liu C.-T., Chen C.-Y., Hsu C.-Y., Huang P.-H., Lin F.-Y., Chen J.-W., Lin S.-J. (2017). Risk of Febuxostat-Associated Myopathy in Patients with CKD. Clin. J. Am. Soc. Nephrol..

[99] Singer J.Z., Wallace S.L. (1986). The allopurinol hypersensitivity syndrome. Unnecessary morbidity and mortality. Arthritis Rheum..

[100] Whelton A., MacDonald P.A., Zhao L., Hunt B., Gunawardhana L. (2011). Renal function in gout: Long-term treatment effects of febuxostat. J. Clin. Rheumatol..

[101] Chohan S., Becker M.A., MacDonald P.A., Chefo S., Jackson R.L. (2012). Women with gout: Efficacy and safety of urate-lowering with febuxostat and allopurinol. Arthritis Care Res. (Hoboken).

[102] Jackson R.L., Hunt B., MacDonald P.A. (2012). The efficacy and safety of febuxostat for urate lowering in gout patients >= 65 years of age. BMC Geriatr..

[103] Wells A.F., MacDonald P.A., Chefo S., Jackson R.L. (2012). African American patients with gout: Efficacy and safety of febuxostat vs allopurinol. BMC Musculoskelet. Disord..

